# Using the theory of planned behavior to predict parents’ disclosure of donor conception to their children: a longitudinal study

**DOI:** 10.1093/humrep/deae070

**Published:** 2024-04-09

**Authors:** Johan Paulin, Kimmo Sorjonen, Gunilla Sydsjö, Claudia Lampic

**Affiliations:** Department of Psychology, Umeå University, Umeå, Sweden; Department of Clinical Neuroscience, Karolinska Institutet, Stockholm, Sweden; Division of Obstetrics and Gynaecology, Faculty of Health Sciences, Department of Clinical and Experimental Medicine, Linköping University, Linköping, Sweden; Department of Psychology, Umeå University, Umeå, Sweden; Department of Public Health and Caring Sciences, Uppsala University, Uppsala, Sweden

**Keywords:** disclosure, the theory of planned behavior, oocyte donation, sperm donation, donor conception, longitudinal study, assisted reproduction, cox regression

## Abstract

**STUDY QUESTION:**

Can the application of the theory of planned behavior (TPB) help predict heterosexual parents’ disclosure of donor conception to their children?

**SUMMARY ANSWER:**

Parents with a stronger will to act in accordance with social norms favoring disclosure were more likely to start the disclosure process within the next 5–9 years.

**WHAT IS KNOWN ALREADY:**

In contrast to single mothers by choice and same-sex couples, heterosexual couples need to make an active decision to disclose their use of donor conception to their child. While disclosure at an early age is encouraged by international guidelines, many heterosexual-couple parents struggle with this. A previous study has found an association between parental scores of TPB factors and disclosure intention, but so far, no study has applied the TPB to predict parents’ disclosure behavior.

**STUDY DESIGN, SIZE, DURATION:**

The present study is based on the fourth and fifth waves of data collection (T4 and T5) in a nation-wide longitudinal study. Participating parents had conceived through identity-release oocyte donation (n = 68, response rate 65%) and sperm donation (n = 62, response rate 56%) as part of a heterosexual couple.

**PARTICIPANTS/MATERIALS, SETTING, METHODS:**

The present study is part of the prospective longitudinal Swedish Study on Gamete Donation (SSGD). Consecutive recruitment of couples starting oocyte or sperm donation treatment was conducted at all seven fertility clinics providing gamete donation in Sweden during a 3-year period (2005–2008). Participants were requested to complete postal surveys at five time points. The present study includes heterosexual-couple parents following oocyte or sperm donation who participated at the two latest time points when their children were 7–8 years old (T4), and 13–17 years old (T5). At T4, participants completed the study-specific TPB Disclosure Questionnaire (TPB-DQ) measuring attitudes and intentions to disclose the donor conception to the child, and disclosure behavior was assessed at both T4 and T5. Data from those participants who had not yet disclosed at T4 were analyzed using survival analysis with Cox regressions.

**MAIN RESULTS AND THE ROLE OF CHANCE:**

Forty participants had not disclosed the donor conception to their children at T4 and, out of these, 13 had still not disclosed at T5. We found a significant association between scores of the TPB factor Subjective norms at T4 and their subsequent disclosure behavior at T5 (HR = 2.019; 95% CI: 1.36–3.01). None of the other factors were significantly associated with disclosure behavior.

**LIMITATIONS, REASONS FOR CAUTION:**

The present study concerns heterosexual-couple parents with children conceived following treatment with gametes from open-identity donors, which limits the generalizability of our findings to other groups and contexts. Other limitations include the risk of systematic attrition due to the longitudinal study design and decreased statistical power due to few participants.

**WIDER IMPLICATIONS OF THE FINDINGS:**

Our findings highlight the importance of perceived subjective norms for parents’ disclosure behavior and indicate that the co-parent’s opinion about disclosure is of particular relevance in this regard. Counselors should focus on supporting prospective parents to initiate and maintain a healthy and open dialogue about concerns around building a family with donor conception.

**STUDY FUNDING/COMPETING INTEREST(S):**

The study was funded by the Swedish Research Council. The authors have no competing interests to declare.

**TRIAL REGISTRATION NUMBER:**

N/A.

## Introduction

In contrast to single mothers by choice and same-sex couples, heterosexual couples need to make an active decision to disclose their use of donor conception to their child. For some, this is an undramatic decision that’s dealt with early on, while for others it’s loaded with much trepidation and hesitation. Comparisons of disclosing and non-disclosing families have shown mixed results, with several studies showing no group differences in family functioning and psychological adjustment (Golombok *et al.*, 2013; Kovacs *et al.*, 2015; Widbom *et al.*, 2022). Other studies, such as longitudinal research from the UK, indicate that early disclosure (before the age of seven) is associated with more positive family relationships and better psychological well-being in families with adolescents (Ilioi *et al.*, 2017) and young adult offspring (Golombok *et al*., 2023). National (The National Board of Health and Welfare, 2004) and international guidelines (Ethics Committee of the American Society for Reproductive Medicine, 2018; Human Fertility and Embryology Authority, 2021; Kirkman-Brown *et al.*, 2022) increasingly encourage parents to start the disclosure process at an early age. Still, parents’ choice of time of disclosure may vary considerably, with some parents intending to disclose early but struggling to find ‘the right time’ (Kirkman, 2003; Daniels *et al.*, 2011; Readings *et al.*, 2011). In addition, the availability of direct-to-consumer DNA tests increases the risk of donor-conceived persons inadvertently finding out about their genetic origins (Crawshaw, 2018). This highlights the need to support parents in their decision to disclose the genetic origin of their child from an early age.

A multitude of factors influencing parents’ decision-making regarding disclosure of the donor conception to their child was synthesized in a systematic review (Indekeu *et al.*, 2013). Intrapersonal factors include stigma associated with infertility and confidence in the use of donor conception, as well as personal beliefs and values about privacy and the best interests of the child/family. Interpersonal factors include couple dynamics and the influence of extended family and friends. Reaching a joint agreement about disclosure within the couple is a complex process with consequences for subsequent disclosure behavior. At the same time, external factors including the role of the socio-cultural-legal context, and the influence of social norms and pressure to conform to traditional family structures also play an important part in the decision process. With that in mind, there is limited knowledge about which factors drive parents’ disclosure intention versus their disclosure behavior, and how to bridge the gap between these.

The theory of planned behavior (TPB) (Ajzen, 1985, 1991) proposes that the intent to perform a specific behavior is the best predictor of the actual performance, and that this intention is predicted by three factors: (i) a person’s attitudes toward the behavior having the desired outcome, (ii) the subjective norms or societal pressure a person perceives to be prevalent regarding the behavior, and (iii) the amount of perceived behavioral control a person has to perform the behavior. The TPB is individually adapted for each research topic and TPB factors have predicted behavior in several different fields and varying contexts, including leaving abusive relationships (Heim *et al.*, 2018), motivations to vote (Barbera and Ajzen, 2020), and attitudes and intentions towards donating oocytes (Purewal and van den Akker, 2010).

In a cross-sectional study, Lampic *et al.* (2021) found that approximately 40% of heterosexual-couple parents following oocyte and sperm donation had not started the disclosure process when the children were 7 years old. Using a TPB instrument tailored for donor conception disclosure (TPB-DQ), they found that parents’ belief that disclosure would have desired consequences and their wish to comply with social norms favoring disclosure, were associated with their intention to disclose the donor conception to the child within the following year. While these results indicate that the TPB may help determine which factors influence parents’ intention to disclose the donor conception, it is unknown to what extent the parents followed through with their intentions.

The present study follows up on the results by Lampic *et al.* (2021), using data from the two most recent waves of data collection of the multicenter longitudinal Swedish Study on Gamete Donation (SSGD). The SSGD includes consecutive cohorts of couples starting gamete donation treatment at seven Swedish university hospitals during a 3-year period (2005–2008). Participants using oocyte donation or sperm donation have completed postal surveys at five time points (T1–T5), from treatment start until the child had reached adolescence. The aim of the present study was to apply the theory of planned behavior to investigate if heterosexual-couple parents’ attitudes and intentions to disclose the donor conception to their child predict subsequent disclosure behavior. Furthermore, we wanted to investigate if parents’ disclosure behavior was moderated by type of treatment (oocyte or sperm donation), gender of the parent, or the parents’ genetic link to the child.

## Materials and methods

### Participants and procedure

The present study includes SSGD participants who had an adolescent child following treatment with oocyte or sperm donation as part of a heterosexual couple. It is based on data from the fourth and fifth wave of data collection of the SSGD, that is, questionnaires distributed to parents in the year following their child’s seventh birthday (T4) and 13th to 17th birthday (T5). The first child born to a participating SSGD couple following conception in 2005–2010 is considered the target child, and parents were instructed to complete specific items concerning this child (indicated by the child’s year and month of birth). In the case of twins, the twin born first was considered the target child. Parents received individual postal questionnaires with a prepaid envelope, together with a letter explaining the study aims and the voluntary nature of participation. Two reminders were sent to non-responders. Of a total of 215 parents approached at T5 (2022–2023), 73/104 oocyte donation parents (70%) and 67/111 sperm donation parents (60%) participated; the total response rate was 65%. After exclusion of 10 parents who had not fully completed the questionnaire at T4, the final sample for the present study consisted of 130 parents (68 oocyte and 62 sperm donation), with 96 parents being part of couple dyads (48 families) and the remaining 34 parents representing one family each.

### Instruments and measurements

The Theory of Planned Behavior Disclosure Questionnaire (TPB-DQ) was designed to specifically measure participants’ beliefs and intentions to disclose the genetic origin to the child within the coming year (Lampic *et al.*, 2021). The TPB-DQ was used at T4 and consists of four factors: *Attitudes, Subjective norms, Perceived behavioral control, and Behavioral intention. Attitudes* consist of seven statements about behavioral beliefs (e.g. ‘I am respecting my child’s rights by talking to him/her about his/her genetic origin.’) and seven accompanying outcome evaluations (e.g. ‘To respect the rights of my child is…’) with responses ranging from ‘Extremely undesirable’ to ‘Extremely desirable’. These items are combined pairwise with a high total score indicating a strong belief that disclosure will lead to a desirable outcome. *Subjective norms* consist of four normative beliefs (e.g. ‘The message from society/media is that I (shouldn’t—should) talk to my child about his/her genetic origin.’), and four items measuring the motivation to comply (e.g. ‘To act according to the messages from society and the media is important to me.’). These items are also combined pairwise with a high total score indicating a strong desire to adhere to perceived societal opinions and norms favoring disclosure. *Perceived behavioral control* consists of six items where a high total score indicates a strong belief in the ability to perform the behavior, in this case disclosure (e.g. ‘I am convinced that I would be able to talk to my child about his/her genetic origin if I wanted to.’). The three items of *Behavioral intention* measure the intent to disclose within a year (e.g. ‘I intend to talk to my child about his/her genetic origin during the coming twelve months’), with high scores indicating a strong intent.

Disclosure behavior was assessed both at T4 and T5 with the study-specific question ‘Have you talked with your child that he/she has been conceived through donation treatment?’. Response alternatives were ‘Yes, I have started talking about it’; ‘No, I intend to do it later on’; ‘No, I intend to do it if/when the child raises the question’; ‘No, I am uncertain/hesitant’; and ‘No, I will not tell the child about this’. For this study, responses were categorized into a dichotomous Yes/No variable, with the four ‘no’ responses combined into one. Parents responding that they had started to disclose were asked to indicate the child’s age when they started (open-response format). Each answer was manually inspected and when the parent had not stated a specific age, variables were replaced as follows: an age range (e.g. ‘8–9 years old’) was replaced by the mean (e.g. ‘8.5 years’), an imprecise response indicating early disclosure (e.g. ‘Early/As long as I can remember’) was replaced with the age ‘1 year’, and the response ‘At birth’ was replaced with the age ‘0 years’. When the response was positive but non-descript (‘Don’t remember/?/Unsure’) we compared if the co-parent of the child had given a more exact age and, if so, that age was used. For the cases where children had not been told by T5, the current age—as of 2023—of the child was used.

### Data analysis

Comparative analysis of characteristics and response rates between oocyte- and sperm-recipient groups were performed using χ^2^ tests. The longitudinal data were analyzed using survival analysis with Cox regressions. The regressions included those parents who answered the TPB-DQ questionnaire, as well as the T5 survey, and who had not disclosed to their child at T4. The number of years elapsed from the child’s birth until disclosure (failure) was used as the time scale, and disclosure as the failure event. Results of Cox regressions were reported in the form of hazard ratios, that is, the ‘risk’ that parents would have disclosed before T5, with 1.0 expressing no added risk, and a result above 1 an increased risk. Hazard ratios account for the time factor by giving an early failure event more weight than a failure occurring later (when the child is older). The total scores of the four factors of TPB-DQ (Attitudes, Subjective norms, Perceived behavioral control, Behavioral intention) were standardized into *Z*-scores for easier comparisons. To illustrate the cumulative effect of disclosure over time, survival analyses were performed with each TPB-DQ factor categorized into three groups (low; moderate; high) based on standard deviations of the *Z*-scores (>1 SD below the mean, ±1 SD, >1 SD above the mean). The use of Cox regression as an analytical method enabled us to estimate how the ratio of parents disclosing to their children related to the TPB-DQ factors, as well as if this was affected when adjusting for possible confounders. All analyses were performed using SPSS 20.0.1.1.

### Ethics

The project was approved by the Regional Ethical Review Board in Linköping, Sweden (M29/05; M29/05/1-06; 2013/299-31) and the Swedish Ethical Review Authority (2022-03739-01).

## Results

### Participant characteristics

Participants included 68 parents following oocyte donation and 62 parents following sperm donation, the majority of which were women. At T5, the mean age of parents was 50.84 (SD = 4.4) and the mean age of the donor conceived children was 15.30 (SD = 1.2). At both time points, most participants reported living with the co-parent of the donor conceived child (T4: 92%; T5: 78%) and having disclosed the donor conception to the child (T4: 69%; T5: 90%). Chi-Square Goodness of Fit tests between donation groups were performed and found a significant difference in disclosure at T5 [χ^2^ (1) = 6.04, *P* = 0.014]. For more detailed characteristics, see Table 1.

**Table 1.** Characteristics of participating parents following oocyte donation and sperm donation.

	Oocyte donation parents		Sperm donation parents	
	Women N (%)	Men N (%)	Women N (%)	Men N (%)
	38 (56)	30 (44)	37 (60)	25 (40)
Occupation T5^a^				
Working	37 (97)	29 (97)	37 (100)	25 (100)
Other	1 (3)	1 (3)	0	0
Relationship status with other co-parent T5^a^				
Married/co-habiting	29 (76)	24 (80)	29 (78)	20 (80)
Divorced/Separated	9 (24)	6 (20)	8 (22)	5 (20)
Disclosure to child T4^b^				
Yes	28 (74)	18 (60)	27 (73)	17 (68)
No	10 (26)	12 (40)	10 (27)	8 (32)
Disclosure to child T5^a^				
Yes	32 (84)	25 (83)	36 (97)	24 (96)
No	6 (16)	5 (17)	1 (3)	1 (4)

a At time of child’s 13th–17th birthday.

b At time of child’s seventh birthday.

The majority of both women and men (76% and 70%, respectively) who participated at T4, also responded to the survey at T5. To investigate potential attrition bias, we compared responders and non-responders at T5 regarding their partner and disclosure status at T4, with separate analyses for women and men. In comparison to the women who responded at T5, the women who dropped out were to a higher extent divorced/separated from the co-parent at T4 (8% versus 29%) [χ^2^ (1) = 7.14, *P* = 0.008] and had not disclosed the donor conception to the child at age 7–8 (27% versus 58%) [χ^2^ (1) = 8.09, *P* = 0.004].

### Association between disclosure beliefs and intention (TPB-DQ) among parents who had not told their child at age 7–8, and their subsequent disclosure behavior

A total of 40 participants had not disclosed the donor conception to the child at age 7–8 (T4). Among these participants, about half (n = 22) were part of a parental couple that responded with regard to the same child. To investigate the relationship between the parents’ scores of the four TPB-factors at T4 and the age at disclosure of the children assessed at T5, we analyzed the data using Cox proportional hazards regressions with years until disclosure (starting from the birth of the child) as time scale and disclosure as the failure event. The results are presented as Hazard Ratios (HR) signifying the ‘risk’ that an event will occur, in this case that a parent will disclose the donor conception to the child. Means and standard deviations of the TPB-DQ factors are presented in Table 2, showing moderately high mean scores for Attitude (m = 35.0, range 1–49), moderately low mean scores for Subjective norms (m = 20.8, range 1–49), and moderate scores for Perceived behavioral control and Behavioral intention, respectively (m = 4.7, m = 4.3; range 1–7).

**Table 2.** Mean and standard deviation of Theory of Planned Behavior-Disclosure Questionnaire (TPB-DQ) score for participating oocyte donation and sperm donation parents at the child’s seventh birthday (T4).

	Oocyte donation parents mean (SD)		Sperm donation parents mean (SD)		Total mean (SD)
TPB-DQ factors	Women	Men	Women	Men	
Attitudes	36.0 (5.76)	34.1 (5.05)	35.8 (5.67)	33.8 (6.18)	35.0 (5.85)
Subjective Norms	23.5 (10.35)	16.8 (6.44)	20.8 (6.97)	22.6 (7.66)	20.8 (8.22)
Perceived Behavioral Control	4.7 (1.08)	4.7 (0.92)	4.8 (1.06)	4.5 (0.94)	4.7 (1.21)
Behavioral Intention	4.2 (1.20)	4.3 (1.49)	4.4 (1.49)	4.2 (1.18)	4.3 (1.35)

Results of the unadjusted Cox regressions using *z*-transformed TPB-DQ scores (Table 3) show that only the TPB-DQ factor Subjective norms was statistically significantly associated with time of disclosure (HR = 2.019; 95% CI: 1.36–3.01). This means that the more the parents reported that they wanted to act according to perceived societal norms favoring disclosure at T4, the more likely they were to have disclosed to their child at T5. In order to account for dependence between couples who answered questions about the same child, we ran a Cox mixed-effects analysis, with respondents grouped within children (1 or 2 respondents for each child). The association between Subjective norms and Disclosure remained statistically significant (HR = 2.245; 95% CI: 1.41–3.58).

**Table 3.** Standardized, unadjusted Cox regressions of the associations between factors of the Theory of Planned Behavior-Disclosure Questionnaire (TPB-DQ) score and disclosure of the donor conception to the child.

TPB-DQ factors	Hazard ratio (95% CI)	Sig (*P*)	Cases available (censored)	Events (N)
Attitude	1.465 (0.96–2.23)	0.074	37 (9)	28
Subjective Norms	2.019 (1.36–3.01)	<0.001	37 (9)	28
Perceived Behavioral Control	1.099 (0.75–1.60)	0.625	38 (10)	28
Behavioral Intention	1.369 (0.94–1.99)	0.098	40 (10)	30

**Figure 1.**
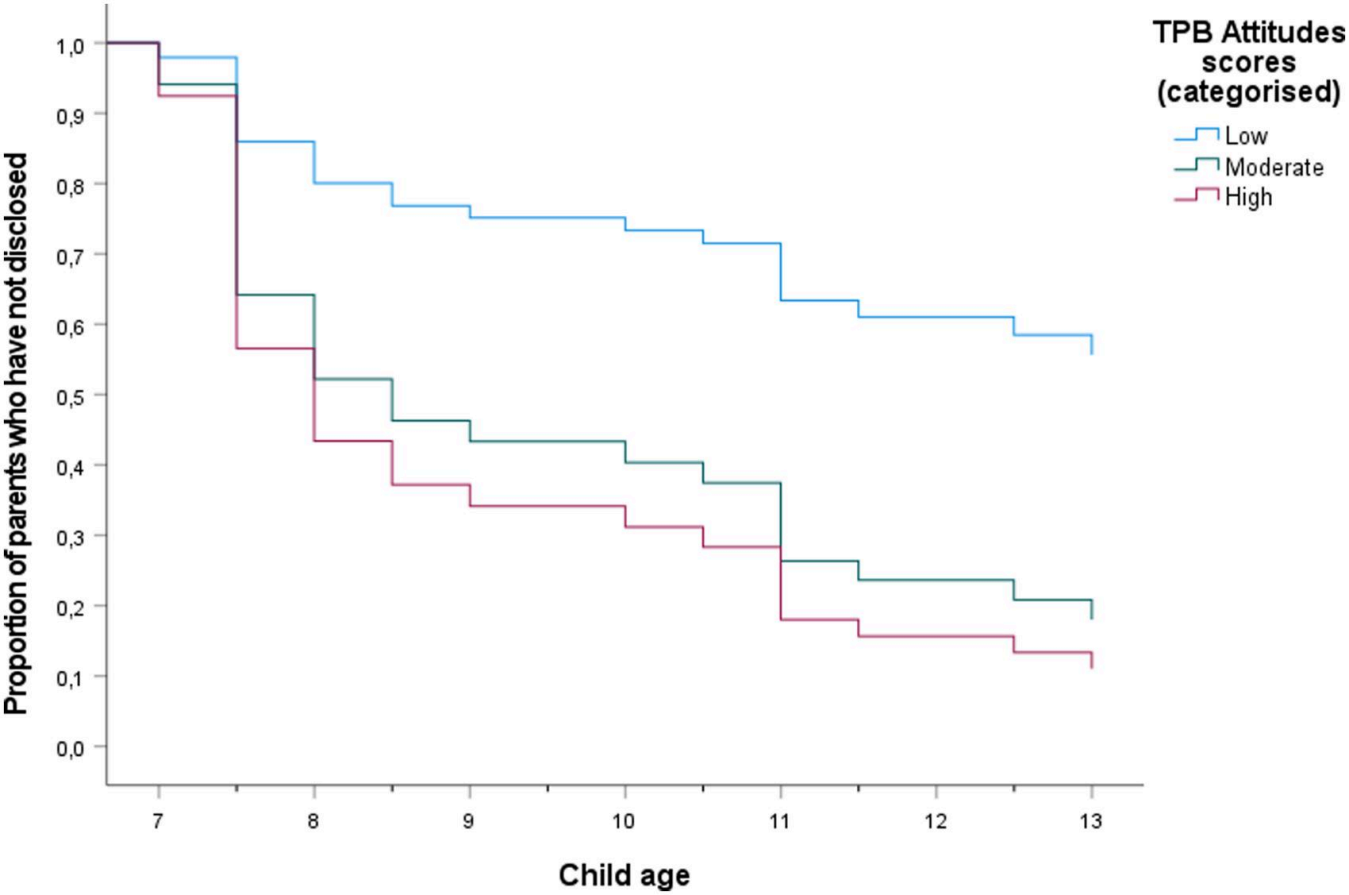
**Survival function of parent–child disclosure over time, determined by TPB-DQ factor Attitude at T4. The Attitude score is divided into three categories.** TPB-DQ: Theory of Planned Behavior-Disclosure Questionnaire.

**Figure 2.**
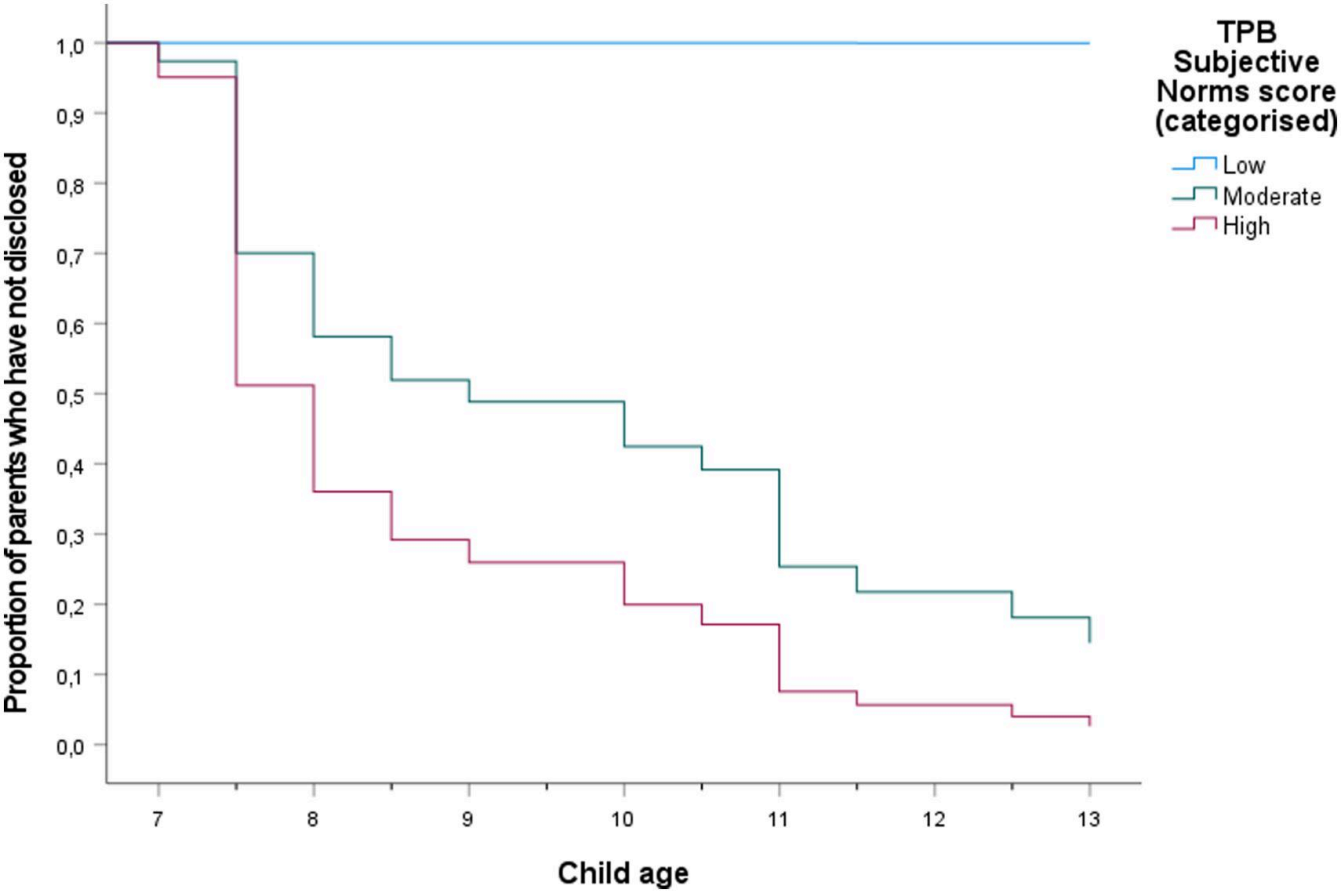
**Survival function of parent–child disclosure over time, determined by TPB-DQ factor Subjective norms at T4.** The Subjective norms score is divided into three categories. TPB-DQ: Theory of Planned Behavior-Disclosure Questionnaire.

**Figure 3.**
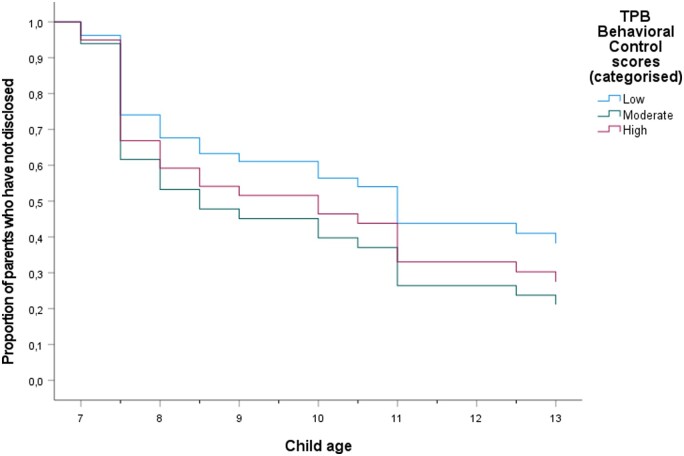
**Survival function of parent–child disclosure over time, determined by TPB-DQ factor Behavioral control at T4.** The Behavioral control score is divided into three categories. TPB-DQ: Theory of Planned Behavior-Disclosure Questionnaire.

**Figure 4.**
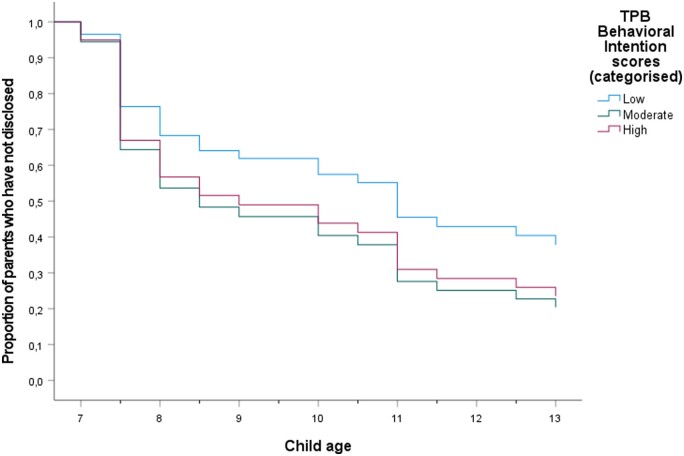
**Survival function of parent–child disclosure over time, determined by TPB-DQ factor Behavioral intention at T4.** The Behavioral intention score is divided into three categories. TPB-DQ: Theory of Planned Behavior-Disclosure Questionnaire.

To illustrate the effect over time, we categorized the *z*-scores for each TPB-DQ factor into three groups (low; moderate; high). Each categorized TPB-DQ factor was then plotted as graphs with Time (child’s age at disclosure) on the *x*-axis and Cumulative survival (number of parents who have not yet disclosed for a given year) on the *y*-axis (Figs 1, 2, 3, and 4). The significant association between Subjective norms and disclosure is illustrated in Fig. 2. None in the group with the lowest scores (n = 5) had disclosed the donor origins to their child up until T5, whereas the cumulative survival (i.e. not disclosing to the child) progressively decreased over time for the medium (n = 25) and high-scoring (n = 7) groups.

Based on the significant effect found on the unadjusted Subjective norms factor, we investigated the potential effect of Subjective norms on parents’ disclosure behavior, while adjusting for parents’ gender, type of donation, and genetic link to the child (gender × donation). Additional hierarchical Cox regressions were performed using these variables as covariates. The four models are presented in Table 4. The adjusted Cox regressions displayed significant associations for Subjective norms (*P* = 0.003). To further explore the effect of Subjective norms we inspected the means and standard deviations for individual items of this factor (Table 5). The highest mean scores were found for the two items measuring normative beliefs and motivation to comply with the respondent’s partner, reflecting the relative importance of this item-pair for the participants’ ratings of the Subjective norms factor.

**Table 4.** Standardized, adjusted Cox regressions of the associations between the Theory of Planned Behavior-Disclosure Questionnaire (TPB-DQ) score and disclosure of the donor conception to the child.

TPB-DQ factors	Hazard ratio (95% CI)	Sig (*P*)	Cases available (censored)	Events (N)
Attitude	1.395 (0.90–2.17)	0.142	37 (9)	28
Sex	0.665 (0.46–9.52)	0.763		
Group	0.987 (0.07–13.77)	0.992		
Sex * Group	1.371 (0.28–6.61)	0.694		
Subjective Norms	2.086 (1.29–3.38)	0.003	37 (9)	28
Sex	2.932 (0.19–44.24)	0.437		
Group	2.736 (0.20–36.62)	0.447		
Sex * Group	0.530 (0.11–2.68)	0.442		
Perceived Behavioral Control	1.066 (0.73–1.57)	0.743	38 (10)	28
Sex	0.630 (0.06–7.28)	0.711		
Group	1.015 (0.09–11.43)	0.991		
Sex * Group	1.484 (0.33–6.75)	0.610		
Behavioral Intention	1.493 (1.00–2.22)	0.049	40 (10)	30
Sex	0.958 (0.09–10.40)	0.972		
Group	1.977 (0.19–20.59)	0.568		
Sex * Group	1.067 (0.25–4.57)	0.931		

Parents’ sex, donor recipient group and genetic link to the child (interaction between sex and recipient group) used as covariates.

**Table 5.** Mean and standard deviation of individual items of the Subjective Norms factor from the Theory of Planned Behavior-Disclosure Questionnaire (TPB-DQ) for participating parents following oocyte donation and sperm donation at T4.

	Oocyte donation parents mean (SD)		Sperm donation parents mean (SD)		Total mean (SD)
Subjective Norms^a^	Women (n* *=* *38)	Men (n* *=* *30)	Women (n* *=* *37)	Men (n* *=* *25)	N = 130
Normative beliefs					
The message from society/media is that I (shouldn’t—should) talk to my child about his/her genetic origin.	6.2 (1.20)	4.9 (1.38)	6.2 (1.13)	5.3 (1.27)	5.7 (1.36)
Most of the people that I feel are important to me think that I (shouldn’t—should) talk to my child about his/her genetic origin	5.5 (1.52)	4.6 (1.32)	5.4 (1.36)	5.7 (1.27)	5.3 (1.42)
My partner thinks that I (shouldn’t—should) talk to my child about his/her genetic origin	6.3 (1.29)	6.1 (1.49)	6.3 (1.22)	6.6 (.88)	6.3 (1.25)
Other couples with donor-conceived children (don’t talk—talk) to their children about their genetic origin	4.8 (1.46)	4.3 (1.52)	4.4 (1.24)	4.8 (1.35)	4.6 (1.38)
Motivation to comply					
To act according to the messages from society and the media is important to me	3.2 (1.96)	2.3 (1.63)	3.0 (2.07)	3.2 (1.58)	2.9 (1.87)
To act according to what people that are important to me feel is right is important to me	3.8 (2.01)	2.5 (1.60)	3.1 (1.82)	3.8 (1.62)	3.4 (1.84)
My partner’s opinion of what I should do is important to me	5.7 (1.63)	5.6 (1.21)	5.8 (1.44)	5.7 (1.27)	5.7 (1.41)
To act in the same manner as other parents with donor-conceived children is important to me	3.1 (1.99)	1.8 (1.26)	2.1 (1.32)	2.9 (1.50)	2.5 (1.64)

a Subjective norms consist of two sub-categories of indirect measures; Normative beliefs and Motivation to comply.

### Application of identified associations between Subjective norms (TPB-DQ) and disclosure behavior to hypothetically predict disclosure behavior among non-respondents at T5

Given the significant association between Subjective norms and disclosure behavior for those who responded at T5, we calculated the hypothetical disclosure rate for T5 non-responders based on their T4 TPB-DQ scores. By using Subjective norms as a predictor of hypothetical disclosure behavior, we performed logistic regression on participants who had not disclosed at T4 but responded at T5. We then used this intercept and coefficient on the participants who had not disclosed at T4 and not responded at T5 to calculate a probability factor. According to this model, the probability of disclosure was similar for responders (75.8%) and non-responders at T5 (78%).

## Discussion

The present study applied the theory of planned behavior (TPB) to investigate if heterosexual-couple parents’ attitudes and intentions toward talking with their child about his/her donor conception could predict parents’ subsequent disclosure behavior. Our results show that parents with a stronger will to act in accordance with social norms favoring disclosure were more likely to start the disclosure process within the next 5–9 years. These results are in line with our previous findings of associations between Subjective norms and the intention to disclose (Lampic *et al.*, 2021), and confirm the importance of this factor for parental disclosure.

The Subjective norms factor of the TPB-DQ measures a parent’s will to comply with perceived norms about disclosure in society at large, as well as among other DC parents, people in one’s close network, and one’s partner. The importance of societal norms and values for parents’ disclosure intentions and behavior has been found in previous research, as reported by Indekeu *et al.* (2013). Inspection of individual item means in the present study revealed the highest scores for the items concerning the partner, who most often was the co-parent of the donor-conceived child. This indicates that participants largely perceived their partner as advocating disclosure and regarded their partner’s opinion as very important. Parents’ agreement regarding disclosure has been investigated in several studies (Indekeu *et al.*, 2013), with some results indicating that a high level of within-couple agreement is associated with parental disclosure.

While our results from a previous study showed that parents’ ratings of the TPB-DQ factor Attitudes were associated with the intention to disclose (Lampic *et al.*, 2021), they were not significantly associated with subsequent disclosure behavior in the present study. Thus, beliefs that disclosing to the child would be the right thing to do and have positive consequences may act as motivating factors, but do not appear to be driving factors for actual disclosure. Furthermore, although uncertainty about how to talk to the child is often highlighted as an important obstacle for disclosure (Indekeu *et al.*, 2013; Applegarth *et al.*, 2016; Hershberger *et al.*, 2021), the present results show no significant associations between Perceived behavioral control and subsequent disclosure behavior, suggesting that feeling confident about disclosing may not translate into carrying through with the act.

Statistical significances were slightly reduced for all TPB-DQ sub-factors when the covariates sex, donation group, and genetic link were added to the analysis (Table 5). The exception being the effects of Behavioral intention and donation group on disclosure which became significant when adjusting for each other (*P* = 0.049). However, as the crude effects of Behavioral intention and group on disclosure were non-significant (*P* = 0.098), we refrain from drawing any substantial conclusions from the adjusted effects.

Parents’ disclosure intention, assessed when the child was 7–8 years old, was not significantly associated with their subsequent disclosure behavior during the following 5–9 years. One possible explanation for this result is that the Behavioral intention in the TPB-DQ in two of three items was expressed as planning to disclose ‘during the coming 12 months’. To our knowledge, only two previous studies have investigated the relationship between parents’ disclosure intentions and behavior with a longitudinal design and both concern oocyte donation families. Hershberger *et al.* (2021) followed six oocyte donation families from pregnancy and found that only one of the three families who initially had intended to disclose had done so by the time the child was 12 years old. On the other hand, Lysons *et al.* (2023) included 73 oocyte donation families when their children were infants and found that a majority of mothers intended to disclose. Among those mothers who initially planned to disclose, just over half had started the disclosure process by the time the children were 5 years old and remaining mothers most often planned to tell the child later (Lysons *et al.*, 2023). More longitudinal studies investigating parents’ disclosure intentions and subsequent behavior are needed to increase understanding of the processes at play.

The present results regarding Subjective norms remained constant even when adjusting for parents’ sex, donation type, and presence or absence of a genetic link to the child and confirm the importance of Subjective norms for heterosexual-couple parents’ disclosure intentions (Lampic *et al.*, 2021) and behavior. If we look at the macro level, this suggests that a societal normalization of family building with gamete donation is desired. Such a reframing of norms needs to be addressed in both clinical guidelines and information provided to recipient couples, as well as in the public discourse where positive role models, open conversations, and debates can increase the perceived societal support. Additionally, positive attitudes and perceived norms can be strengthened by providing long-term psychological and clinical aftercare for recipient couples, in part to provide support for them, but also to address thoughts and questions that may arise post-conception (Kirkman-Brown *et al.*, 2022).

The main methodological strengths of the present study are the longitudinal design and the application of the theory of planned behavior to predict disclosure behavior. A weakness common for longitudinal studies is the risk of systematic drop out over time. While we achieved relatively high response rates at T5 for parents following oocyte donation (70%) and sperm donation (60%), attrition analyses show that drop out was significantly more likely among mothers who were divorced/separated from the child’s father, and mothers who had not started the disclosure process at T4. However, our hypothetical prediction model found an equally high disclosure probability among responders and non-responders, which may suggest that attrition was not related to disclosure issues, but rather due to some other unknown factor(s). Another limitation is low statistical power in our main analyses due to the small number of participants who were non-disclosers at T4, which may explain why the present results did not completely align with our previous results (Lampic *et al.*, 2021). Finally, our sample of heterosexual couples undergoing treatment with gametes from open-identity donors limits the generalizability of our findings.

## Conclusion

Despite national and international recommendations that parents should talk with their child about his/her donor conception from an early age, many heterosexual couples struggle with the process of disclosure (Mac Dougall *et al.*, 2007; Applegarth *et al.*, 2016). A key finding of the present study is the importance of perceived subjective norms for parents’ disclosure behavior, in particular the importance of the co-parent’s opinions. In addition, our results do not provide support for the common notion that parents fail to talk with their child because they are uncertain about when and how to disclose. Our results suggest that counselors should focus on supporting prospective parents in initiating and maintaining a healthy and open dialogue about thoughts and concerns around building a family with donor conception. By doing so, it might be easier for parents to talk with their children about the donor conception not just early, but continuously throughout childhood. This is also in line with the wishes expressed by donor-conceived children (Schrijvers *et al.*, 2019). The present study contributes new theory-based insights into factors affecting disclosure behavior, which need to be tested in other samples and contexts in order to enable international comparisons as well as deepen the knowledge base around disclosure processes.

## Data Availability

The data underlying the article will be shared on reasonable request to the corresponding author.
